# IL-1β Antibody Protects Brain from Neuropathology of Hypoperfusion

**DOI:** 10.3390/cells10040855

**Published:** 2021-04-09

**Authors:** Dominic Quintana, Xuefang Ren, Heng Hu, Deborah Corbin, Elizabeth Engler-Chiurazzi, Muhammad Alvi, James Simpkins

**Affiliations:** 1 Department of Neuroscience, School of Medicine, West Virginia University, Morgantown, WV 26506, USA; ddquintana@mix.wvu.edu (D.Q.); xuren@hsc.wvu.edu (X.R.); hehu@hsc.wvu.edu (H.H.); drcorbin@hsc.wvu.edu (D.C.); eenglerchiurazzi@tulane.edu (E.E.-C.); 2 Center for Basic and Translational Stroke Research, Department of Neurology, Rockefeller Neuroscience Institute, School of Medicine, West Virginia University, Morgantown, WV 26506, USA; muhammad.alvi@hsc.wvu.edu

**Keywords:** IL-1β, IL-1β antibody, canakinumab, cerebral hypoperfusion, white matter damage and grey matter damage

## Abstract

Chronic brain hypoperfusion is the primary cause of vascular dementia and has been implicated in the development of white matter disease and lacunar infarcts. Cerebral hypoperfusion leads to a chronic state of brain inflammation with immune cell activation and production of pro-inflammatory cytokines, including IL-1β. In the present study, we induced chronic, progressive brain hypoperfusion in mice using ameroid constrictor, arterial stenosis (ACAS) surgery and tested the efficacy of an IL-1β antibody on the resulting brain damage. We observed that ACAS surgery causes a reduction in cerebral blood flow (CBF) of about 30% and grey and white matter damage in and around the hippocampus. The IL-1β antibody treatment did not significantly affect CBF but largely eliminated grey matter damage and reduced white matter damage caused by ACAS surgery. Over the course of hypoperfusion/injury, grip strength, coordination, and memory-related behavior were not significantly affected by ACAS surgery or antibody treatment. We conclude that antibody neutralization of IL-1β is protective from the brain damage caused by chronic, progressive brain hypoperfusion.

## 1. Introduction

Chronic cerebral hypoperfusion is the primary cause of vascular dementia [1] and has been implicated in the development of white matter disease and lacunar infarcts [2]. Further, brain hypoperfusion predicts the rate of cognitive decline in patients with Alzheimer’s disease and the occurrence of dementia in mild cognitive impairment (MCI) patients [3,4,5]. During the progression of brain hypoperfusion, there is metabolic distress and bioenergetic disturbance that contribute to cerebral degeneration [6].

Though the pathophysiology resulting from chronic cerebral hypoperfusion is complex, a key factor underlying the degenerative mechanistic cascade may be neuroinflammation. Indeed, numerous reports have noted a chronic state of brain inflammation [7] with immune cell activation [8] and production of pro-inflammatory cytokines [9] including IL-1β [10,11]. The potential role of pro-inflammatory cytokines in brain damage from hypoperfusion is suggested by the observation of a cytokine storm after stroke [12,13,14] and the efficacy of the IL-1β antibody, canakinumab, in stroke protection [15]. However, the role of IL-1β in brain damage associated with chronic cerebral hypoperfusion has not been examined.

In the present study, we used a mouse model of cerebral hypoperfusion [16,17], by applying ameroid constrictor arterial stenosis (ACAS) surgery to induce chronic, progressive brain hypoperfusion and tested the effects of repeated administration of an IL-1β antibody on behavioral and neuropathology outcomes.

## 2. Materials and Methods

### 2.1. Animals

Male C57BL/6 J mice, 12~14 weeks old, were obtained from the Jackson Laboratory. Mice were housed in accordance with Institutional Animal Care and Use Committee (IACUC) guidelines of the West Virginia University (WVU) Health Sciences Center vivarium and were maintained under a light/dark cycle (12:12 h) with food and water available ad libitum. All performed procedures were approved by IACUC of West Virginia University.

### 2.2. Implantation of Ameroid Constrictor Ring and Microcoil

The ameroid constrictor is a device used in veterinary medicine for the treatment of hepatic shunts that induces collateral circulation via blood vessel occlusion. The constrictor ring is composed of a surgical steel ring and an inner layer composed of casein, the hygroscopic property of which causes its gradual swelling at a predictable rate. The surgical steel ring that surrounds the casein layer forces this swelling inward, resulting in a shrinking inner diameter that gradually occludes blood vessel.

Mice were randomized to sham surgery and ameroid constrictor, arterial stenosis (ACAS) surgery groups. Mice were anesthetized with 4–5% isoflurane and maintained under 1–2% isoflurane in a 30% O2:70% N2 mixture and placed on a feedback-controlled heating pad to maintain the body temperature at 37 °C. Ophthalmic ointment was placed on the eyes; the surgical area was prepared by trimming the fur and sanitizing the skin with isopropanol pads followed by betadine. Both common carotid arteries (CCAs) were exposed through a midline cervical incision, and an ameroid constrictor ring (cat. MC-0.50-55, Research Instruments SW, Escondido, CA, USA was placed around the right CCA following a published protocol [16,17]. A microcoil (cat. SWPA ID 0.18, Wuxi Samini Spring Co., Ltd., Jiangsu, China) was placed around the left CCA to prevent cerebral blood flow (CBF) compensation. The entire surgical procedure for each animal was completed within 20 min. The control group received a sham surgery including an incision exposing CCAs, but neither the ameroid constrictor rings nor the microcoils were implanted. All further experimentation was performed blinded to surgical group.

### 2.3. Administration of IL-1β

The highly specific IL-1β-surrogate anti-mouse IL-1β antibody (01BSU) was provided by Dr. Hermann Gram (Novartis, Basel, Switzerland). The IL-1β antibody used is a selective IL-1β monoclonal antibody [18] and is similar to canakinumab, clinically used for a number of autoimmune and inflammatory conditions [19,20]. A murine IgG2a/k isotype was used an isotopic control, as in other studies [21]. 01BSUR is specific for mouse IL-1β (KD 302 pM) and does not bind to IL-1α and IL-1β receptor [22].

**Figure 1:**
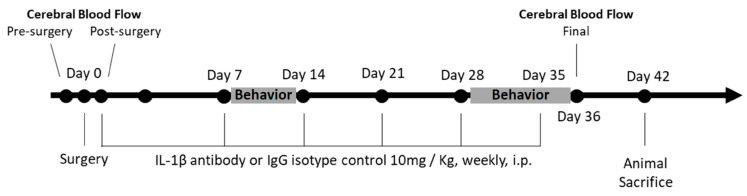
Schematic of the experimental design for the assessment of the effects of hypoperfusion and an IL-1β antibody on behavior and neuropathology in mice.

Sham and ACAS surgery mice were randomized to IgG (Isotopic Control) or IL-1β antibody treatment groups; resulting in 4 experimental groups with n = 9 mice/group: sham surgery + IgG, sham surgery + IL-1β antibody, ACAS + IgG, and ACAS + IL-1β antibody. Both IgG and IL-1β antibody were administered at a dose of 10 mg/kg body weight, i.p., weekly, with the first dose given immediately after the sham or ACAS surgery, and the last dose on post-surgery day 35. The experimental protocol is depicted in Figure 1.

### 2.4. Cerebral Blood Flow

Before the placement of the ameroid constrictor ring and microcoil, baseline CBF was measured. Briefly, the skull was exposed with a 1.5 cm incision. Ten consecutive measurements of CBF were acquired with a MoorFLPI laser Doppler system (Moor Instruments, Axminster, Devon, UK) over the course of 5 min with the exposure of 200 ms. To ensure that the surgery procedure itself did affect blood flow to the brain, a second CBF measurement was performed right after the implantation. The incision was closed with sutures, and the animals were subcutaneously injected with a local anesthetic, bupivacaine (1 mg/kg), once per day for 3 days. Subsequent CBF measurements were performed on day 36 by reopening the scalp at the same incision site, followed by suturing and bupivacaine injections.

### 2.5. Behavioral Assessment

Behavioral assessments of all animals were conducted on days 8–13 and 29–36 after ACAS or sham surgeries, similar to previously published procedures with slight modifications [16,23,24]. In brief, all animals were assessed in the following order for grip strength, rotarod coordination, and Y-maze and water radial arm maze cognition-related behavior tests. For grip strength measurements, we utilized a commercial grip strength meter with the grid attachment employed (Ugo Basile, Gemonio, Italy) to acquire data on grip force (gf), total pull duration (s), and time of peak force (s). Data were collected over a series of three trials, with each trial comprised of three successful pulls; a successful pull was defined as a subject grasping the grid with both paws on unique grid squares at the start of the pull; if a mouse failed to meet these criteria on a given pull, data from that pull were discarded. For rotarod testing, we utilized a commercial apparatus (Ugo Basile, Gemonio, VA, Italy) set to accelerate from 4–40 rpm over 300 s and conducted 3 trials within one day with a 5 min inter-trial interval, similar to what we have carried out previously with minor modifications. Spontaneous alternation and recognition ability were assessed using a Y-maze similar to how others have implemented it. Animals were placed individually in one arm of the three-armed apparatus and allowed to freely explore for 8 min. Arm entry sequences were tracked using the commercially available software, Any Maze (Stoelting, Chicago, IL, USA), and the percent of successful alternations, defined as entry into the least most recently visited arm within a three-arm entry sequence (i.e., A-B-C vs. A-B-A), was determined. Spatial working and reference memory were assessed using the two-day water-based radial arm maze test following the program developed by Alamed and colleagues [23] with some modifications. For all trials, a single hidden platform was submerged approximately 1 cm below the surface at the end of one of the maze arms; the water was made an opaque grey color with non-toxic tempera dye to obscure the platform and aide in software tracking of the mouse (Anymaze, Stoelting, Chicago, IL, USA). For all trials, the location of the hidden escape platform remained fixed across all days and trials for a given subject and at a given timepoint post-injury; animals were tested on two groups of six trials, with each grouping separated by approximately 2 h (total of 12 trials per day) each for two days. Spatial cues (geometric shapes or patterns with high contrast to the background) to aid in navigation were hung from curtains surrounding the maze and remained in fixed locations for all days and trials across both timepoints. Visual ability was verified using the visible platform task, in which a black colored platform with a flag was placed just above the water’s surface at the end of a single arm; grey-colored water was made opaque with non-toxic dye as in hidden platform test days but extramaze cues were removed and animals were tested on 6 trials on a single test day. For both hidden and visible platform tests, errors (entries into arms that did not contain the escape platform), as well as swim distance, trial duration, and swim speed were collected.

### 2.6. Histochemistry

On day 42, mice were anesthetized and transcardially perfused with 20 mL of 0.01 M phosphate buffered saline followed by 20 mL of 4% paraformaldehyde, pH of 7.45. Brains were extracted, placed in 4% paraformaldehyde, and incubated at 4 °C overnight. Fixed brains were sliced into 2 mm coronal sections, embedded in paraffin, sliced into 10 μm coronal sections, and mounted onto specimen slides. Deparaffinized and rehydrated tissue sections were stained with haemotoxylin and eosin (H&E) to assess general brain pathology [25] and silver stained to assess axonal damage [26]. The sections were imaged on an MIF Olympus VS120 Slide Scanner at 20× magnification.

All categories and severity of grey and white matter injuries were scored (See Table 1) and summed for each animal. Then the summed scores for each group and their variances were calculated and are reported.

### 2.7. Statistical Analysis

Statistical comparisons were performed using ANOVA (with or without repeated measures, where appropriate). Dunnett’s post-hoc test was used for comparison of the experimental groups relative to a control group, or for comparison within a given group at one time point post-surgery (sham or surgical hypoperfusion) relative to the pre-surgery time point. *p* < 0.05 was considered significant.

## 3. Results

### 3.1. Body Weight and Mortality

Animals assigned to each of the four groups were not different in body weight at the baseline (prior to surgery or treatment). Final body weights were not different between sham and ACAS groups with or without antibody treatment. All sham + IgG, sham + IL-1β antibody, and ACAS + IL-1β antibody mice survived until the day of planned sacrifice (day 42). Three of the ACAS + IgG mice died, one each on days 10, 14, and 16 post-surgery.

### 3.2. Cerebral Blood Flow

**Figure 2:**
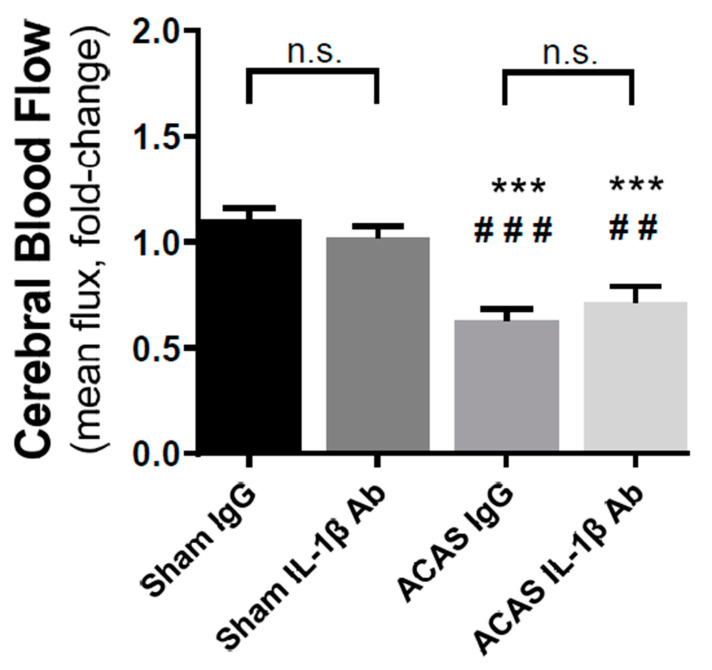
Effects of ameroid constrictor, arterial stenosis (ACAS) surgery and IL-1β on cerebral blood flow 36 days after ACAS or sham surgery and the initiation of IL-1β antibody or IgG treatment. N = 9 mice/group; *** *p* < 0.001 vs. sham IgG; ^###^ *p* < 0.001 vs. sham IL-1β Ab. n.s. = not significantly different.

Cerebral blood flow (CBF) was assessed prior to surgery and treatments, then again on day 36 post-surgery. There were no group differences in CBF prior to surgery. Depicted in Figure 2 are the changes from baseline (pre-surgery) in CBF. In sham surgery animals, IgG or IL-1β antibody treatment did not change CBF (F = 14.94, *p* = 0.7335). In ACAS surgery animals, which received IgG, CBF was reduced by 37% (F = 14.92, *p* < 0.0001) from baseline, while ACAS animals treated with IL-1β antibody showed a 29% (F = 14.94, *p* < 0.009) decline from baseline in CBF. These differences were not significant.

### 3.3. Grey Matter Damage

**Figure 3:**
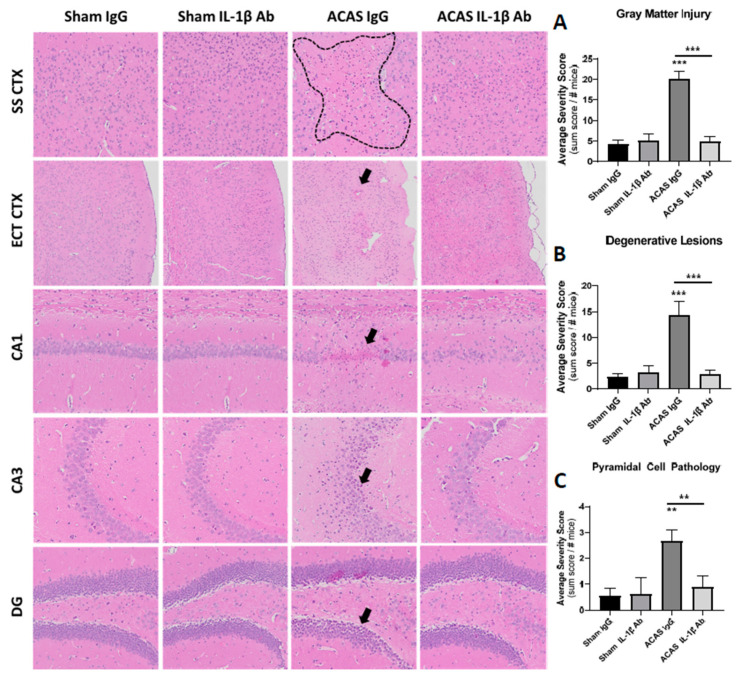
Grey matter pathology at 42 days after ACAS or sham surgery and the initiation of IL-1β antibody or IgG treatment. Depicted are the four treatment groups: sham + IgG, sham + IL-1β Ab, ACAS = IgG, and ACAS + IL-1β Ab for the following brain regions: SSCTX = somatosensory cortex; ECTCTX = entorhinocortex; CA1 and CA3 = regions of the hippocampus, and DG = dentate gyrus of the hippocampus. Areas shown with dotted lines or arrows are examples of lesions that were quantified as shown in Table 1. Dashed lines in the SSCTX outline a degenerative lesion; the arrow in the ECT CTX indicates a condensed lesion; the arrows in the CA1, CA2, and DG show cell loss, Bar graphs shown in (**A**–**C**) are mean ± SEM for lesion severity scores for each of the four treatment groups. N = 9 mice/group. ** *p* < 0.01 vs. the sham + IgG group or the ACAS + IgG group. *** *p* < 0.001 vs. sham + IgG group or the ACAS + IL1-β Ab group.

Grey matter damage was scored by a blinded observer based on the observation of any of four types of damage in brain slices: degenerative lesions, pyramidal cell loss, granular cell loss, or vacuolizations (Table 1). As each lesion identified was scored, there was no upper limit in the sum score for grey matter damage. As shown in Figure 3, the sum scores for the sham surgery controls were low and not different with IgG or IL-1β antibody treatment (F = 24.88, *p* = 0.73). ACAS resulted in a 4.7-fold (F = 24.88, *p* < 0.0001) increase in sum score for grey matter damage in the IgG group (Figure 3). In contrast, the ACAS group receiving IL-1β antibody, did not show an increase in grey matter damage in comparison (F = 24.88, *p* = 0.92) to the sham surgery group receiving IL-1β antibody (Figure 3).

### 3.4. White Matter Damage

**Figure 4:**
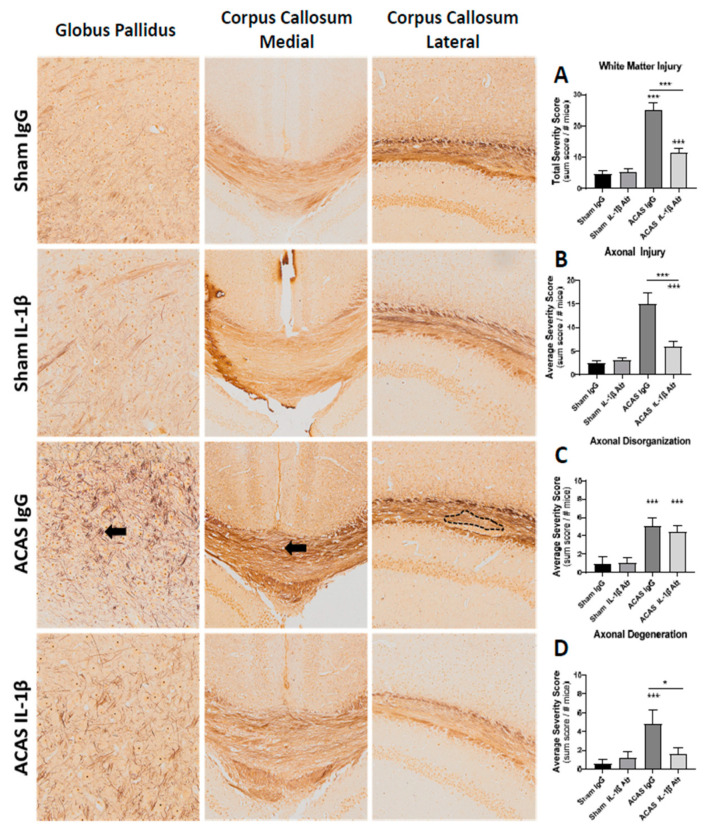
White matter pathology at 42 days after ACAS or sham surgery and the initiation of IL-1β antibody or IgG treatment. Depicted are the four treatment groups: sham = IgG, Sham + IL-1β Ab, ACAS = IgG and ACAS + IL-1β Ab for the following brain regions: globus pallidus and corpus callosum. The arrow in the ACAS IgG globus pallidus slide shows disorganized axons; the arrow in the ACAS IgG corpus callosum medial slide shows axonal loss; and the dashed line in the ACAS IgG corpus callosum lateral slide shows axonal disorganization. Bar graphs shown in (**A**–**D**) are mean ± SEM for lesion severity scores for each of the four treatment groups: *n* = 9 mice/group; * *p* < 0.05 between the connected groups; *** *p* < 0.001 vs. sham + IgG group or the ACAS + IL1-β Ab group.

White matter damage was scored by a blinded observer based on axonal injury, axonal disorganization, and degeneration (Table 1). As shown in Figure 4, all three indices of axonal injury were significantly increased in the ACAS + IgG group versus the sham + IgG group. Similarly, in all cases the ACAS + IL-1β group was reduced versus the ACAS + IgG group.

### 3.5. Behavioral Outcomes

Among the test conducted, no significant differences between surgery group, with or without IL-1β, were observed at either 8–13 days or 29–36 days (data not shown).

## 4. Discussion

The present study demonstrates that brain hypoperfusion induced by ACAS surgery results in brain damage, which is effectively eliminated by chronic treatment with the IL-1β antibody, a potent pro-inflammatory cytokine produced and released by multiple immune cells in both the brain and periphery [27,28]. With progressive hypoperfusion, brain damage can result from activation of brain microglia or peripheral immune cells. After acute hypoperfusion such as that which occurs after stroke, an early increase in blood and brain neutrophils is seen [27,28,29]. IL-1β antibodies may neutralize this source of IL-1β, effectively reducing the impact of brain invading neutrophils. Alternatively, IL-1β antibodies may cross the compromised blood-brain barrier (BBB) during hypoperfusion and neutralize brain-released IL-1β released from microglia or invading immune cells. In either case, chronic neutralization of IL-1β appears to provide protection of the brain from the neurodegenerative effects of chronic hypoperfusion.

It is well known that microglia are the prominent innate immune cells in the brain and participate in neuroinflammation in neurodegenerative diseases [30]. Microglia respond to pathological stimulations by producing pro-inflammatory cytokines, such as IL-1 beta [31]. The changes in microglia have not been evaluated in the ACAS model yet; however, it is possible that IL-1beta antibody has a protective effect against microglia activation, which has been demonstrated in other neurodegenerative diseases [32].

The profound effects of the IL-1β antibody on neuropathology during chronic hypoperfusion is similar to the magnitude of effects that we have observed with blood replacement following acute hypoperfusion cause by stroke in mice [29]. In the latter case, we observed that blood replacement reduced peripheral and brain neutrophils, and thereby reduced secretion of pro-inflammatory cytokines, such as IL-1β [29]. This raises the possibility that IL-1β, while not effecting entry of neutrophils into the brain, may reduce brain damage by reducing the impact of secreted IL-1β. Peripheral administration of an IL-1β receptor antagonist is protective in both transient and permanent middle cerebral artery occlusion models of stroke [33,34,35], and the IL-1β antibody, canakinumab, was shown to be protective in strokes in a clinical trial [15].

The lack of effects of ACAS or the IL-1β antibody on grip strength, rotarod coordination, or cognition-related behavioral outcomes in our study is not surprising to us for several reasons. First, the brain damage from ACAS surgery was sparse (Figure 3 and Figure 4) and likely too small to cause overall strength, coordination, or cognition-related behavioral dysfunction. In this regard, in mice with large infarcts post-stroke, while motor function deficits can be documented, it is difficult to detect cognitive behavioral effects [36,37] and when they are seen, they are typically subtle [38]. Importantly, the study population tested here were healthy, young adult, male mice, a population who may be resilient to functional deficits caused by subtle neuropathological changes induced by the ACAS surgery. Thus, the resulting hypoperfusion in these young adult animals may need to persist for a longer period of time to observe behavioral decline in these animals. To date, only a handful of studies have evaluated the long-term consequences of chronic hypoperfusion in these models. Evidence to date suggests that impairments may be evident as early as 28 days and for as long as 6 months following ACAS, bilateral carotid artery stenosis, or gradual common carotid artery stenosis, other surgical induction methods of cerebral hypoperfusion [16,39,40,41]; the degenerative nature of the deficits is of interest given that working memory dysfunction tends to appear earlier in the progression, while later both working and reference memory deficits are evident. Whether age or the presence of underlying comorbidities (i.e., diabetes, hypertension) would exacerbate these cerebral hypoperfusion deficits has not yet been well studied [42,43]. Taken together, the lack of significant behavioral effects in our study population is encouraging and suggests that otherwise healthy organisms may likely experience limited functional deficits from hypoperfusion, at least initially, thus providing an opportunity to target effective preventative interventions. However, given that vascular dementia, the human medical condition that ACAS models, is an age-related disease that often develops over the course of many decades and manifests in the presence of a number of modifiable and non-modifiable comorbidities, the evaluation of how the presence of these comorbidities interact to influence the trajectory of functional decline, as well as the potential for inflammatory modulators to affect this trajectory, will be an important area of future study.

Our study has different effects on the CBF from those of Maher et al. [4] who reported a profound decrease in CBF in rats with IL-1β and a more modest increase in CBF with an IL-1ra. The differences could be due to species differences but are more likely due to the route of administration of the compounds (ICV vs. IP), the use of an IL-1ra versus an antibody, and the continuous [44] versus episodic (our study) exposure to the compound.

The present study has several limitations. First, we cannot rule out white matter edema as a cause for white matter damage. Although we report degenerative lesions in mice that received hypoperfusion, we cannot distinguish the cause of degenerative lesion/white matter injury, such as via edema, hypoxia, and inflammatory changes. Second, the area of the brain assessed for damage from hypoperfusion was limited to the hippocampus and surrounding cortical regions. As such we cannot be sure that the damage induced by hypoperfusion or the effects on IL-1β antibody are generalizable to the whole brain. Third, a single dose of IL-1β antibody (10 mg/kg, i.p.) was administered once every seven days and we do not know if this dose or dosing interval is optimal. The dosing paradigm used was based on the observation of a terminal half-life of canakinumab of 17.7 days when administered at a dose of 10 mg/kg in mice [45]. Finally, the hypoperfusion induced was profound and occurred over a short time period, in contrast to that seen in human subjects with milder hypoperfusion over decades of life.

## Figures and Tables

**Table 1:** Tissue Scoring and Scale.

**Grey Matter Damage**	
**Score**	**Degenerative Lesion**
1	Anomolous structure
2	Necrotic tissue
3	Hole or tissue atrophy

**Score**	**Pyramidal Cells Loss**
1	Irregular cell layer
2	Thinning of cell layer
3	Gap in cell layer

**Score**	**Granular Cells Loss**
1	Apoptotic cells
2	Thinning of stratum granulosum
3	Gap in stratum granulosum

**Score**	**Vacuolization**
1	Sparse vacuoles
2	Moderate vacuoles
3	Dense vacuoles
**White Matter Damage**	
**Score**	**Axonal Injury**
1	Sparse axon damage
2	Moderate axon damage
3	Dense axon damage

**Score**	**Axonal Disorganization**
1	Majority of axon bundles retained
2	Moderate retention of axon bundles
3	Majority of axon bundles lost

**Score**	**Axonal Degeneration**
1	Sparse tortuous axons
2	Moderate tortuous axons
3	Dense tortuous axons

## Data Availability

The data from this study are in the electronic files at the WVU.
